# Patient and public involvement in international research: Perspectives of a team of researchers from six countries on collaborating with people with lived experiences of dementia and end‐of‐life

**DOI:** 10.1111/hex.13942

**Published:** 2023-12-24

**Authors:** Shirin Vellani, Marie‐Lee Yous, Vanessa Maradiaga Rivas, Stephanie Lucchese, Julia Kruizinga, Tamara Sussman, Julia Abelson, Noori Akhtar‐Danesh, Gina Bravo, Kevin Brazil, Rebecca Ganann, Sharon Kaasalainen

**Affiliations:** ^1^ School of Nursing, Faculty of Health Sciences McMaster University Hamilton Ontario Canada; ^2^ School of Social Work, Faculty of Arts McGill University Montreal Quebec Canada; ^3^ Department of Health Research Methods, Evidence and Impact, Faculty of Health Sciences McMaster University Hamilton Ontario Canada; ^4^ Department of Community Health Sciences, Faculty of Medicine and Health Sciences Sherbrooke University Sherbrooke Canada; ^5^ School of Nursing and Midwifery Queens University Belfast Northern Ireland UK

**Keywords:** dementia, end‐of‐life, interpretive description, palliative approach, patient and public involvement, reflexive thematic analysis, research engagement

## Abstract

**Background:**

Patient and public involvement (PPI) is a critical priority in research, policy, academia and advocacy organizations. PPI in dementia research is gaining momentum. However, these efforts are missing in international projects aimed at those living with advanced dementia in long‐term care (LTC) homes. Additional complexities can arise in enacting PPI within the context of integration of a palliative approach to care and experiences around end‐of‐life in (EOL) dementia. The mySupport study involved implementing the Family Carer Decision Support (FCDS) intervention for care partners of those living with advanced dementia in LTC in six countries.

**Research Design and Objective:**

An interpretive description study was conducted to explore the perspectives of international researchers from six countries on engaging people with lived experiences of dementia and EOL care in research processes. The findings from this study informed the development of a PPI strategy and a subsequent toolkit for the FCDS intervention.

**Findings:**

Thirty‐eight interviews were completed with project researchers: 12 from the United Kingdom, 8 from Canada, 7 from Ireland, 4 each from Italy and The Netherlands and 3 from the Czech Republic. Four broad themes describe international researchers' perspectives on advancing methods of engagement for people with lived experiences of dementia and EOL in international PPI activities: (1) Groundwork to engage in research; (2) planning for research activities is key; (3) focus on meaningful engagement and (4) having foresight for practical issues shaping PPI.

**Discussion and Implications:**

International projects that involve PPI can present many sources of challenges. The findings in this study highlight important considerations for foundational work for incorporating PPI in international projects. Learning from world leaders and those with lived experiences in various regions can be insightful and help share tools and resources.

**Patient or Public Contribution:**

PPI was envisioned as a critical part of conducting the mySupport study. The findings from this study informed the development of a PPI strategy and an international Strategic Guiding Council that included family carers of those living with advanced dementia in LTC homes in six countries. This manuscript focused on the perspectives of researchers on their engagement with people with lived experiences of dementia and EOL. The perspectives of persons with lived experiences on engaging in the mySupport research study will be reported in a forthcoming manuscript.

## 1 INTRODUCTION

Patient and public involvement (PPI) in research and knowledge dissemination activities is broadly accepted as a best practice in health and social care research, as it improves the quality of research, promotes transparency and ensures relevance.^1^, ^2^, ^3^ It refers to engaging patients, care partners and other members of the public, such as community leaders, in the design, execution and dissemination of research.^3^ PPI can range from involvement in public dissemination plans to complete partnerships in both managing the research process and implementing change.^4^ PPI is a critical priority for funding bodies, policymakers, researchers, academic journals and various advocacy organizations^3^, ^5^ including those representing people with dementia and their care partners.^6^

PPI in dementia research is gaining momentum. For example, the Canadian Dementia Priority Setting Partnership involved individuals with lived experiences of dementia, that is, persons living with dementia and their care partners, and successfully identified top 10 dementia research priorities.^7^ The Canadian Consortium on Neurodegeneration in Aging (CCNA) Engagement of People with Lived Experience of Dementia Advisory Group has demonstrated active participation of people with lived experiences as partners in the CCNA research process.^8^ Also, the Scottish Dementia Working Group Research Subgroup entirely comprised of people living with dementia, created core principles and resources to engage people with dementia in research.^9^ More recently, a UK‐based research team involved people with lived experiences of dementia as study advisors in all stages of a randomized controlled trial of a psychosocial intervention, ‘Journeying through Dementia’.^10^ Though they experienced challenges to ensure meaningful engagement due to constraints related to time and resources, they reported improved research accessibility and relevance.

The above initiatives are promising; however, PPI efforts that are international in scope, that centre care partners of persons with advanced dementia, and that are initiated in complex health environments such as long‐term care (LTC) or nursing homes are rarely represented in the literature. This paper redressed this gap in the literature by reporting on researchers' experiences engaging with care partners supporting persons at EOL with advanced dementia in LTC.

The mySupport study is an international multidisciplinary implementation study to support family carers of persons with advanced dementia living in LTC homes, in making complex decisions surrounding EOL care.^11^ The study was conducted in Canada, the Czech Republic, Ireland, Italy, The Netherlands and the United Kingdom. PPI was envisioned as a critical part of conducting the mySupport study and the findings from the current qualitative study informed the development of a PPI strategy which included the formation of an international Strategic Guiding Council.

The Strategic Guiding Council, which was formed after these interviews took place, comprised of family care partners of those living with advanced dementia in LTC homes in five countries (unable to recruit from Italy), recruited virtually early during the COVID‐19 pandemic and hosted by the Canadian research team members. The council was established in October 2020 beginning with an individual virtual orientation session. Each member was then assigned a research buddy from their own country to support them with their activities, translation and assist with providing written feedback to the larger group. The council met virtually quarterly to discuss study updates, provide feedback on study materials through structured activities and share upcoming opportunities to participate in. Their perspectives on engagement in the mySupport study is presented in another manuscript which is currently prepared for submission. Details of PPI strategy in the context of mySupport study including how the groups were formed, their composition, role and communication exchange; as well as; blogs, videos and a podcast can be found on the official study website, https://mysupportstudy.eu/ and in previous publications.^12^, ^13^ As such, the purpose of this qualitative study was to acquire international perspectives from the project investigators to inform a public engagement plan that accommodates the unique cultural context of each participating country. As well, explore their perspectives on engaging persons with lived experiences of dementia and EOL care in the research processes and knowledge dissemination activities.

## 2 METHODS

### 2.1 Design

The research design of this qualitative study was Thorne's interpretive description approach.^14^ It involves revealing the subjective and experiential knowledge of participants to inform applied practice when engaging in research with persons with lived experiences. Interpretive description was most suitable for this study as it focuses on commonalities of experiences and multiple constructed realities that may conflict at times,^14^ thus allowing diverse perspectives to be shared. Ethics approval to conduct this study was obtained from the Hamilton Integrated Research Ethics Board: 2019‐5837‐GRA, McMaster University. All participants provided written informed consent to participate. COnsolidated criteria for REporting Qualitative (COREQ) checklist is used to report this study to optimize transparency in reporting the study methods in turn improve the credibility and comprehensiveness^15^ (see Supporting Information S1: Appendix B for the COREQ checklist).

### 2.2 Participants and setting

Participants consisted of investigators, with various roles within academic institutions and organizations, from six countries in Europe and North America who were part of the mySupport study. Every researcher in the mySupport study was invited to participate and provide rich descriptions of their experiences in engaging in research with people with lived experiences.^10^, ^12^ Individual interviews were conducted virtually and scheduled at the convenience of participants.

### 2.3 Data collection

Semistructured individual interviews were conducted by one of the three research assistants from the Canadian mySupport research team in the Summer and Fall of 2019. They received training in qualitative research interviews. All interviews were conducted over the phone and recorded using Zoom communication software and ranged in length from 30 to 45 min. The interview guide was developed by the Canadian mySupport research team members with expertise in qualitative research, dementia, and palliative and EOL care research and informed by a review of the literature for concepts related to PPI in research (see Table 1). It was pilot‐tested with another researcher before conducting interviews with international researchers. Interview audio recordings were transcribed by a professional transcriptionist and reviewed for accuracy by two research assistants from the Canadian mySupport research team.

**Table 1:** Interview guide.

‐ What experience, if any, have you had involving the public or patients in your research? ‐ Describe your experiences of leading/implementing a study that includes the patients, public or partnering organizations within a research study ‐ What parts of the research were they involved in? ‐ What are your opinions or views on how patient engagement works in research? ‐ Based on your experience, what are the downsides to involving patients in research ‐ What do you think about the prospect of involving patients in the mySupport study? ‐ What do you think about involving care partners in the study? ‐ What do you think about involving partner organizations in this study such as the Alzheimer's Society? ‐ How prepared do you feel to work with patients and care partners on this project ‐ What kind of training do you think would be helpful for this? ‐ Thinking about the mySupport study, do you think there will be barriers related to patient engagement within this project ‐ What is your opinion related to working with different countries in this project?

### 2.4 Data analysis

Reflexive Thematic Analysis (RTA) was employed in this study as it well aligned with Thorne's interpretive description approach.^14^ RTA is a theoretically flexible approach to qualitative data analysis that recognizes a researcher's active role in the identification and analysis of patterns and themes generated to meet the objectives of a research study.^16^, ^17^ We focused on generating experiential themes that would support a comprehensive understanding of participants' views, experiences and feelings regarding PPI. We used the six phases of thematic analysis to guide our analytic process.^18^ More specifically our team engaged in a process of (a) gaining familiarity with the data; (b) coding; (c) searching for initial themes; (d) reviewing themes; (e) developing a name and definition for themes and (f) completing a written report.^18^ Rather than linearly impose these steps, our team approached these stages in a recursive and iterative manner.

In the initial stages of coding, five different transcripts were independently read and coded by five members of the research team (S. V./V. M. R./S. K./J. K./S. L.). Following this process, all five researchers collectively discussed their assumptions and interpretations of the data in an effort to generate an initial coding structure to guide subsequent analysis. While S. V. and V. M. R. continued to code the remaining transcripts, ongoing team reflections and discussions helped to refine and reshape themes. For example, while prerequisites for PPI, considerations for cultural diversities, the role of various organizations and developing a research team were initially conceptualized and named as separate themes, our collective reflections and discussions led us to consider the ways in which cultural diversities, organization as a partner and team development could be framed as prerequisites for international PPI initiatives. We, therefore, combined these initial themes and renamed the prerequisite with groundwork to better reflect the essence of the reconceptualized theme and associated sub‐themes (see Supporting Information S1: Appendix C for an example of the initial coding tree). Our team used the qualitative programme Dedoose as a coding tool as this web‐based programme allows for multiple users.^19^ In line with the interpretive description approach, rigour and trustworthiness were maintained throughout the data analysis process.^14^

### 2.5 Overview of findings

A total of 38 interviews were completed with project researchers from the mySupport study research team: 12 from the United Kingdom, 8 from Canada, 7 from Ireland, 4 each from Italy and The Netherlands and 3 from the Czech Republic. In terms of their role in the mySypport study, 7 were principal investigators, 22 were co‐investigators, 4 were partners or collaborators from various organizations such as All Ireland Institute of Hospice & Palliative Care and Alzheimer Society Canada and 5 were research staff on the team.

Except for one, all participants had previously engaged in some form of research activity involving people with lived experiences of dementia and EOL care. Table 2 provides an overview of participants' experience engaging people with lived experiences based on Health Canada's five‐level PPI framework.^4^

**Table 2:** Former experience of PPI engagement by level.

	Health Canada's Public Involvement Continuum					
Countries	Level 1	Level 2	Level 3	Level 4	Level 5	No experience
Total (*n* = 38)	Inform or educate	Gather information	Discuss (two‐way information exchange)	Engage (PPI may influence decisions affecting them)	Partner (PPI throughout the research and KTE process)	
Canada	1		1	3	3	
United Kingdom	1			1	10	
The Netherlands	1		1	2		
Czech Republic	1	1				1
Ireland	1		3		3	
Italy		4				

Abbreviations: KTE, knowledge translation and exchange; PPI, patient and public involvement.

Regardless of their former experience and comfort in involving individuals with lived experiences, most agreed that engagement of people with lived experiences is critical in research activities because it ‘will make research more relevant and more grounded’ (Participant 11). Researchers also felt that ‘organizations, services; health, social and family care can gate‐keep and restrict access to people with dementia’ (Participant 8).

Alongside participants' overall endorsement of the importance of PPI in health and social care research, participants expressed a series of considerations regarding engaging people with lived experiences within international projects. As such, four broad themes describe international researchers' perspectives on how best to advance the methods of engagement for people with lived experiences of dementia and EOL, in international research and knowledge dissemination activities: (1) Groundwork to engage in research; (2) planning for research activities is key; (3) focus on meaningful engagement and (4) having foresight for practical issues shaping engagement of people with lived experiences. Details of the themes and subthemes are presented below with a summary presented in Table 3. Supporting Information S1: Appendix A presents additional illustrative quotes for each of the subthemes below.

**Table 3:** Themes and subthemes.

Themes	Subthemes
1‐ Groundwork to engage in research, people with lived experience of dementia and EOL care	A‐ Advocacy for engaging experts by experience
	B‐ Consideration for cultural diversities across countries
	C‐ Partnership with organizations
	D‐ The carefully cultivated research team
2‐ Planning for research activities is key	A‐ Thoughtful process of engaging people with lived experiences
	B‐ Consideration for accessibility and accommodation
	C‐ Remunerate for time, knowledge and expertise
3‐ Focus on meaningful engagement	A‐ Involve with purpose and avoid tokenism
	B‐ Garnering trust in the research relationship
	C‐ Strive to create guidance for future research engagement
4‐ Having foresight for practical issues shaping engagement of people with lived experiences	A‐ Awareness for vested interests of diverse people
	B‐ Watch out for the emotional needs and vulnerabilities of partners
	C‐ Mitigate challenges of retention

Abbreviation: EOL, end‐of‐life.

#### 2.5.1 Groundwork to engage in research

Groundwork to engage in research, people with lived experiences of dementia and EOL care describes the importance of efforts to ensure strategic and coordinated PPI and to enhance the research team's capacity to undertake international projects with PPI as a major component. Specifically, the groundwork to engage in research involves appreciating the value of PPI and advocating for its need in research processes, building relationships with advocacy organizations in each country and cultivating a research team.

1. *Advocacy for engaging experts by experience*: Several participants highlighted the importance of being advocates for engaging people with lived experiences in research and integrated knowledge translation activities to leverage their expertise based on their experiences. All participants attested to the value of engagement to make sure research is relevant to people and seeks their active input in its design, conduct and dissemination. Many highlighted that there remains a ‘paternalistic distinction’ (Participant 10) in dementia research leading to the exclusion of people with lived experiences of dementia and EOL in PPI. Hence, participants shared the importance of advocating for PPI by showcasing their past successes on academic, political, and social forums and to help demystify some challenges. One participant shared:
  There is a new sort of … it's not a conference, but it's being developed over here and it's called Involve Fest and it's basically a PPI festival, basically recognizing the research that has been done to involve all the carers. And I think it just demonstrates that there is a greater awareness now of how important it is to have co‐production, co‐design, to have that PPI, that patient‐centered approach… to be able to demonstrate that we have incorporated PPI throughout and that they have been so important to leading the study and help us disseminate work at the end. (Participant 19)

Participants also shared that the research team needs to be intentional in fostering an environment where people can share their views without influence and ‘coercion’ (Participant 8) from others for it to add the most value to the project. Some participants use the term experts for people with lived experiences, as one researcher explains:

Excluding patients and the public because they weren't experts and how could they get involved, obviously led to really under‐informed and sometimes completely misled research. But their expertise…so I like using the term experts by experience because it's a kind of reminder of why you are engaging this group. They are experts, but they are experts in actual lived experience with the thing that you are trying to understand. (Participant 10)

2. *Consideration for cultural diversities across countries*: As part of the groundwork for PPI, participants also recognized the importance of considering cultural diversities and their impact on international research endeavours. Cultural diversity was highlighted in terms of the team (researchers and people with lived experiences), as well as the varying characteristics of their jurisdictional environments. Most participants expressed cultural diversity as a strength as it brings forth a unique lens, while several were sceptical and concerned about potential challenges in conducting international collaborations. Participants pointed out logistical challenges in bringing people together from various regions in relation to language and time differences, technological challenges and the availability of support that could be provided by the research teams, as well as the differences in teams' level of comfort in engaging people with lived experiences. Participants also noted challenges related to cultural differences that are more high‐level and engrained in societal norms.
  Some participants expressed that the idea of a palliative approach to care and goals of care conversations, particularly about EOL are not usual practices in LTC homes in their region, but that through participation in this study, LTC staff will be allowed to think about these concepts. One participant articulated that,
  I think it can really help the research to be useful at the end. So, I like the idea very much. It's just that in our country it's not very usual to involve patients or families like that, so I look forward to this experience as part of this project. (Participant 24)

Overall, most participants shared that international collaborations can serve as an opportunity to learn from each other and identify best practices for involving persons with lived experiences, and if they could be adapted in their unique contexts.

3. *Partnership with organizations*: Researchers identified that building relationships with various advocacy organizations is quite an important groundwork because many of them have established advocacy and support groups that involve people with lived experiences of dementia. Participants reported that building relationships with service and advocacy organizations can serve to be synergistic. Not only can they serve as liaisons for recruiting people with lived experiences, but they can also participate in executing research when their agendas align with the project and help with the dissemination of findings. One researcher explained:
  It adds credibility to the results if you can have the support of all these organizations and say that the [x organization] has been involved in this research from the start. They helped to direct the research, looked at the results and endorsed the results. I think that adds huge credibility. (Participant 31)
4. *Carefully cultivated research team*: Finally, in laying the groundwork for engaging people with lived experiences in research, participants also underlined the critical importance of cultivating a research team which involved hiring compassionate people and educating them on dementia, different ways people are affected by it, and most importantly, communication strategies. Participants attested that research involving people with lived experiences of dementia and EOL care adds an enormous layer of complexity to the project than engaging people with other chronic medical conditions. Participants also shared that research teams should be sensitive in discussing palliative and EOL issues and develop expertise in facilitating such discussions. Several participants also emphasized the importance of building proficiency in being constructive and collaborative when providing feedback and navigating differences in opinions. Overall researchers articulated several strategies to laying the groundwork for engaging people with lived experiences of dementia and EOL care in research before initiating research activities.

#### 2.5.2 Planning for research activities is key

The second theme highlights the fundamental importance of thorough planning that involves specific considerations to ensure that the PPI activities run efficiently in the context of people with lived experiences of dementia and EOL. Participants highlighted that the important elements in planning involve a thoughtful process for engaging people with lived experiences, consideration for accessibility and accommodation needs, ascertaining jargon is avoided and, ensuring engagement events and materials are not overloaded with information; as well as timely remuneration for people's time, knowledge and expertise.

1. *Thoughtful process of engaging people with lived experiences*: Planning a thoughtful process to engage people with lived experiences was a major subtheme. Participants shared their views about recruiting from an existing pool of people accessible through specific institutions which may be feasible but not ideal and may introduce bias to the studies. They articulated that participation in multiple studies can lead to ‘a researcher fatigue element for them too’ (Participant 30). There was also a concern that PPI activities generally involve those in the early stages of dementia, while people with advanced disease are generally represented by their care partners, whose perspectives may be different than those living with dementia.
  Given the focus of the mySupport study was care partners of those living with advanced dementia in LTC homes, the majority of researchers suggested only including care partners. Researchers expressed concerns about LTC home residents' cognitive and physical capacity for partnering in the research process. One researcher articulated their concerns about recruiting from LTC homes:
  People who are in a care home, if they have capacity, they will be a resident in the care home because they need a lot of physical support, so they are going to become tired easily. I would think just the whole context is pretty challenging. It's everything from the kind of logistics, have you really thought through how much time people have, what's comfortable, where they want to do it, and so on and so forth to conducting it sensitively and ethically and how you deal with challenging issues that come up (Participant 10)

Referring to care partners' engagement, many shared that PPI activities can add to the existing burden associated with caregiving duties, hence a higher likelihood of burnout and dropout from the study. Some participants suggested engaging individuals with past experiences of caring for persons as opposed to those currently involved in caregiving duties. These individuals may better reflect on their experiences and bring consumer viewpoints of the healthcare systems. Regardless of the people's lived experiences, international researchers indicated that it is critical to have a comprehensive orientation and negotiation of the terms of engagement.

2. *Consideration for accessibility and accommodation*: In addition to a thoughtful process of engaging people with lived experiences, planning also involves laying out considerations for accessibility and accommodation needs. Accessibility was discussed in relation to the appropriateness of the physical space, for example, availability of a ramp, elevator, easy‐to‐reach venue and parking. As well as accessibility of documents and materials, which should be jargon free. Participants also acknowledged the importance of having a sense of flexibility when collaborating with persons living with dementia and/or their care partners. For example, meetings should be scheduled based on their availability based on their daytime job, caregiving duties, acute changes in condition and time of the day when the person with dementia may be most energetic and alert. Persons with dementia, particularly those with no prior experience engaging in research activities, may want to participate with a trusted individual such as a family member or friend, and it should be fostered. Participants recognized an important role of the research team where a dedicated staff member can provide ongoing and in‐the‐moment support and serve as a point person who helps with booking appointments, sharing meeting notes and connecting on an individual basis to respond to their queries and arranging for technical support when it involved virtual engagement.
3. *Remunerate for time, knowledge and experience*: In discussing planning to engage people with lived experiences in research, participants stressed the importance of budgeting for fair remuneration promptly, for their time, knowledge, and expertise. There was a consensus among participants that it is not about money or the incentive, remuneration is a display of acknowledgement for their time and input and creates a sense of mutuality. Participants suggested complementary activities such as festivals to recognize the involvement of people with lived experiences in research. Some participants also reported that in their region, funding agencies may evaluate their budget for terms of engagement, and hourly rates for their work and if not optimal, grants may be rejected. Many participants also shared facing dilemmas in allocating different rates for different types of people. For example, one participant explained:

There is a lot of technical difficulty with compensating these people. Does everybody receive the same amount whatever they do? Or whatever group they represent? For example, in my grant application I have older adults, caregivers of people with dementia, but also doctors and nurses. And then you know, is the time of a doctor worth the same thing as the time of an older adult? I think it's an interesting ethical question. My answer to that is yes, they should get the same amount. (Participant 19)

Overall, participants described various important considerations while planning for PPI involving those with lived experiences of dementia and EOL including a careful process of identifying who to involve, considerations for accessibility and accommodation as well as remuneration and other activities to recognize PPI and create further awareness.

#### 2.5.3 Focus on meaningful engagement

The next theme focused on the meaningful engagement of people with lived experiences of dementia and EOL care, in research and knowledge dissemination activities. Participants identified that engagement should not be tokenistic but involve an open appreciation of the value they bring. Efforts should be made to emphasize the strength they bring to the research process while building a trusting relationship. Engagement can serve as an opportunity to develop and enhance guidelines on how best to involve persons living with dementia and their care partners in future research and knowledge dissemination partnerships.

1. *Involve with purpose and avoid tokenism*: Participants consistently highlighted the critical importance of making concerted efforts to engage with purpose and avoiding tokenism. They identified that funding bodies are increasingly demanding to include individuals with lived experiences of dementia in research activities. However, they are included procedurally to fulfil the application requirement, rather than as partners to inform the research processes. One researcher succinctly articulated this:
  If you don't adapt so that people can be meaningfully involved, then they are just there but they are not really having a say… And I'm not sure that they actually go out of their way to see what their view is on something or to orient them to how these meetings work or maybe adapt the meetings to be more friendly towards a family member who maybe doesn't come from the business world and knows how board meetings work. So I've seen places where they are there in name, but not really… (Participant 15)

As such, several participants stressed the need for mindful efforts to actively encourage people with dementia and/or their care partners to share their perspectives and feedback and in some cases, lead the initiative. As a result, there will be a greater potential for influencing change and future collaborations involving people with lived experiences.

2. *Garnering trust in research relationship*: Garnering trust in research relationships is another common subtheme in designing meaningful engagement of people with lived experiences of dementia and EOL care. Several participants recommended rethinking the term ‘patient’ as this term comes from the hospital sector and is suggestive of the medical model as opposed to a person‐centred model and may not be conducive for collaborative projects involving LTC homes. Participants suggested that given dementia and EOL care‐related research is inherently sensitive, research teams should aim to first connect with people at the individual level to build rapport. Participants also specified that research and researchers can come across as complicated and academic which can be ‘off‐putting’ (Participant 3). Therefore, information exchange should be based on people's negotiated capacity while impressing upon them that their input can make research relevant and accessible for the public and those at the endpoint of research projects.
3. *Strive to create guidance for future research engagements*: Several participants conveyed that PPI in the current international project can serve to be an opportunity to co‐design guidelines with input from people with lived experiences. These guidelines can be related to the best ways to involve persons with lived experiences with dementia and EOL care in research and knowledge dissemination activities, as well as, how best to integrate a palliative approach to care with dementia management. Participants reflected that some countries such as the United Kingdom may be far more advanced in stipulating the processes of involving people with lived experiences than other countries. The experiences acquired in the current research can lead to the identification of key messages to bring multinational consistency and coherence regarding the conduct of future research. Participants also acknowledged that this engagement project can also lead to the identification of a definition of effectiveness, a tool, that could be used to assess the effectiveness of engagement to be used across the countries. Overall, participants relayed several strategies and aspirations for meaningful engagement of people with lived experiences ranging from including them as partners and building trust to the creation of guidelines.

#### 2.5.4 Having foresight for practical issues

The final theme is foresight for practical issues which describes how researchers should develop an awareness of the vested interests of diverse people and stakeholders engaged in research activities. As well as be cognizant of people's emotional needs and vulnerabilities by being a person living with dementia or their care partner; and potential challenges related to their retention in research activities that may be longstanding.

1. *Awareness of vested interests of diverse people*: One of the most prominent subthemes related to practical issues was having foresight for various people's vested interests serving as motivations to participate in the research activities which may affect the dynamics of the research process. Some participants shared the importance of being aware of differences in agendas by countries and sometimes by jurisdictions in a region which may not align with the ethos of the research. Also, care partners may have a ‘personal vendetta’ (Participant 26) based on their past experiences with healthcare, that they may want to address by way of engaging in research activities. Several participants expressed the importance of having an awareness of various motivations and setting clear expectations at the outset.
2. *Watch out for emotional needs and vulnerabilities of partners*: Participants were unequivocal about being mindful of the emotional needs and vulnerabilities of people with lived experiences in dementia and EOL care. They also highlighted that these needs are not only related to the sensitive nature of the topics under discussion, but vulnerabilities could also be triggered by power dynamics between various participants such as scholars or businesspeople and people with lived experiences; as well as persons living with dementia and their care partners.
  Concern was shared around family members and sometimes staff (when the study involves LTC homes), who may try to protect their person living with dementia by restricting their full engagement in research activities. Participants noted that when there are power dynamics, generally older adults choose not to speak. They proposed making resolute efforts to obtain buy‐in from family and staff for the project. As well, separate various categories of individuals and frequently check in on people with lived experiences to optimize support for them and their participation in the process.
3. *Mitigate challenges of retention*: Finally, foresight on practical issues also involves looking out for challenges related to retention and mitigating them. Many participants shared that older adults may doubt their ability to participate in research and may feel intimidated by researchers' titles. They need to be ongoingly reminded of the value of their experiences and perspectives in the research activities. Participants also identified challenges of retention in relation to a person losing their cognitive capacity during the course of the study or dying due to frequently having multiple medical conditions.

In terms of care partners, they may also experience challenges in staying engaged as they may be juggling various commitments demanding their time. They may also experience the loss of their person with dementia during the study and go through a process of grieving. Participants also emphasized being cognizant of care partners' feelings of apprehension as dementia progresses and caregiving responsibilities change, which may deter them from continuing engagement in the research activities.

Many participants felt that researchers should try to limit research activities and expectations for time commitment according to people's capacity. Participants acknowledged that some studies may take longer than expected to culminate, and researchers should strive to support people not only during the study period but also connect them to resources for support beyond the study. Overall, international researchers identified multiple issues shaping the engagement of people with lived experiences in dementia and EOL care, in research activities and suggested strategies to anticipate and overcome them.

## 3 DISCUSSION

The current study examined international researchers' perspectives on PPI in international research and knowledge dissemination activities in the context of dementia and EOL care. Perspectives acquired from the project investigators informed the public engagement plan and the development of a Strategic Guiding Council, reflecting the unique cultural context of each participating country. Overall, there was a consensus among researchers that engagement of those living with dementia and/or their care partners is crucial in research and knowledge dissemination. Based on their own past experiences in PPI and learning edges, researchers were able to identify key strategies and requisites to engage persons with lived experiences in dementia and EOL care in research. Essentially, our findings highlight important considerations for foundational work before engaging in international projects incorporating PPI, planning the actual engagement activities, meaningful partnership opportunities for people with lived experiences and pragmatic issues impacting PPI in ongoing and future activities.

PPI involving co‐design efforts with persons with dementia are beginning to emerge particularly for early to moderate stages of the condition and there is some evidence of success with these endeavours.^7^, ^8^, ^10^ Projects aimed at later stages of dementia rarely include a co‐design. Our work fills this gap by exploring researchers' perceptions of engaging care partners of persons with advanced dementia on projects related to advanced stages of dementia including care at the EOL. It is a first step towards the eventual implementation and evaluation of a PPI strategy. Based on the findings of the current study, a Strategic Guiding Council was formulated for the mySupport study that included family carers of those living with advanced dementia in LTC homes in six countries. With the active involvement of care partners, local teams translated and adapted intervention materials according to respective countries' ethical and medicolegal requirements and developed culturally sensitive content. This process and the subsequent adaptations adopted for the Family Carer Decision Support (FCDS) intervention have been reported elsewhere.^11^, ^20^

Findings of this study complement previous work involving the engagement of older individuals with multimorbidity^21^, ^22^; as well as those living with dementia, as research partners.^7^, ^8^ Identifying individuals who are representative of the diverse individuals living with the disease and their care partners and striving to develop a trusting relationship with them while uplifting the capacity of the research team are keys to effective PPI partnerships.^21^ We and others have also established the critical importance of developing a trusting relationship with individuals with lived experiences through respect; and open and skilful communication strategies for mutually fulfilling research partnerships.^7^, ^10^, ^11^ Like Ganann et al.,^22^ our findings also attested to being aware of the potential burden people can experience with participation in research activities. This is in relation to multiple requests for participation, balancing multiple responsibilities and accessibility needs in terms of language, information overload and a feeling of intimidation from academics, businesspeople and policymakers.

Our findings move forward with an understanding of how best to incorporate PPI concerning international projects that engage people with lived experiences of dementia and EOL care, including those living with the condition and their care partners. The findings also highlight international researchers' apprehensions for the successful engagement of people living with advanced dementia in LTC homes, and their care partners given challenges associated with cognitive and physical abilities, logistical issues involving LTC home routines and resources as well as contextual challenges of each participating country. Hence, mySupport study engaged family carers in PPI activities for the international implementation of FCDS intervention.

Evidence is accumulating on how best to engage people with lived experiences in dementia including persons living with dementia and their care partners in research as active partners,^8^, ^23^, ^24^ but it can get more complicated in international endeavours due to cultural nuances. Furthermore, those living with dementia continue to face stigma and discrimination by family, friends, clinicians, the general public and policymakers as to their capacity to meaningfully engage and effectively contribute.^25^ This could be due to a lack of understanding of the disease, and benevolent ageism.^26^ In turn, people with dementia may develop the belief that they cannot contribute to research activities, and it can be difficult to persuade them otherwise.^27^ What is important is to consider the unique needs of support in any vulnerable group ^28^ and tailor approaches to cater to them by utilizing local resources and expertise. International projects can provide opportunities for reflection and growth. They can serve to be eye‐opening and interesting for people coming from different cultures regarding both PPI and dementia to learn from one another, and this can be examined as an outcome in future PPI endeavours.

When the research involves matters related to EOL care in the context of dementia, PPI can present further complexities. No matter how archaic it may sound, death and dying remain taboo subjects in society.^29^ Topics related to EOL, such as identifying a person's wishes and values as well as guiding care partners to learn about the disease process can help with wish concordant care. Yet, these topics sit at the junction of sensitive and contentious where it may be difficult to predict people's response to such discussions. Depending on the culture, people and institutions may not be willing to engage in EOL‐related research and such discussion may cause strong emotional reactions in the moment and people may choose to leave the project. Some researchers suggested including care partners who have gone through the care journey and had a decent time grieving. Some also suggested that international projects like mySupport study can serve as a learning opportunity for those uncomfortable with dementia and EOL‐related topics. Hence, such international PPI projects can be considered both harder and more valuable as they hold the potential for a culture shift. It is important that research teams acknowledge the inherent sensitivity of such topics and convey respect and appreciation to people engaging in the partnership. As well as develop their own capacity for compassionate engagement through interpersonal and communication skills^30^ to be able to recognize where support is needed and what local resources to mobilize, lasting beyond the study period if needed.

It was identified earlier that FCDS intervention was developed to support family care partners of those living with advanced dementia in LTC homes. Perspectives of project investigators informed the implementation of the FCDS intervention with active PPI in diverse regions of Europe and North America. It is critical to keep up the momentum through more PPI projects engaging those residing in LTC homes. Typically, LTC home residents are less frequently involved in research partnerships because a large percentage have physical and cognitive disabilities and many die within a short period of admission in the home.^30^ However, LTC homes are usually the final residence for an individual and therefore future PPI research shall promote a culture change towards a relational person‐centred model from the ever‐existing institutional medical model of care.^31^

Given that LTC homes are complex adaptive systems, it is crucial to build relationships with various stakeholders within the home such as the resident/family council, management, and various categories of staff to foster champions in the LTC homes who can facilitate and liaise with research teams and residents and their care partners. Nonetheless, chronic systemic deficiencies and staffing shortages add multiple layers of complexities in engaging LTC home residents, staff and care partners in research partnership.^32^ As such, PPI involving international studies involving an international group of researchers can be challenging, however, the world has become a global village and therefore opportunities for an international team‐up should be fostered.

## 4 FUTURE DIRECTIONS

In future studies, there is a need to carefully consider the stages that are necessary to support the meaningful engagement of persons with lived experience in research regarding palliative and EOL care in the context of dementia. Reflection is necessary at the outset of research by completing groundwork to gain a better understanding of advocacy initiatives within local organizations and enhance the cross‐cultural dialogue among research teams. Practical issues such as how sensitive topics are discussed should be considered at the start of engaging research partners to enable their retention in study activities over time. Active participation of LTC home residents, including those with dementia, should be prioritized. This would require identifying creative means for PPI to include residents in the later stages of dementia who may not partake in traditional ways, such as art‐based approaches to engagement.^31^, ^33^ Finally, there is a need to develop guidelines for conducting international research in the context of dementia and EOL care, incorporating the component of process evaluation.

## 5 STRENGTHS AND LIMITATIONS

The study had multiple strengths including the inclusion of multiple countries and the large sample size. With regard to limitations, this manuscript only focused on the perspectives of researchers and interviews were conducted in English. Other papers exploring the perspectives of persons with lived experiences in engaging in research are forthcoming. The Canadian research team member conducted interviews in English across different countries. English may not have been the primary language of participants which could have led to possible challenges in being able to fully express themselves. In future studies, interviews could be conducted in multiple languages based on the preferences of participants.

## 6 CONCLUSION

The findings of this study provide many valuable insights concerning the engagement of persons with lived experience in dementia and EOL care as research partners. Our results can serve as a resource for those who want to embark on similar research.

## AUTHOR CONTRIBUTIONS

All authors have provided substantive contribution in the creation of this manuscript and meet the criteria for authorship as stated in the Uniform Requirements for Manuscripts Submitted to Biomedical Journals. Specifically, authors Sharon Kaasalainen and Tamara Sussman have been involved in the design of the study. In addition to authors Shirin Vellani, Sharon Kaasalainen and Vanessa Maradiaga Rivas, other authors Marie‐Lee Yous, Julia Kruizinga, Tamara Sussman, Stephanie Lucchese, Gina Bravo, Kevin Brazil, Noori Akhtar‐Danesh, Rebecca Ganann and Julia Abelson were substantially involved in the acquisition, analysis and interpretation of data and drafting manuscript. Shirin Vellani, Sharon Kaasalainen, Marie‐Lee Yous, Vanessa Maradiaga Rivas, Julia Kruizinga, Tamara Sussman, Stephanie Lucchese, Gina Bravo, Kevin Brazil, Noori Akhtar‐Danesh, Rebecca Ganann and Julia Abelson were involved in critically revising the manuscript for important intellectual content and approval of final submitted version; as well agree to be personally accountable for all aspects of their contribution towards the completion of this manuscript. The authors read and approved the final manuscript.

## CONFLICT OF INTEREST STATEMENT

The authors declare no conflict of interest.

## ETHICS STATEMENT

Ethics approval to conduct this study was obtained from the Hamilton Integrated Research Ethics Board: 2019‐5837‐GRA, McMaster University. All participants provided written informed consent to participate.

## Supporting information


*Appendix A. Additional illustrative quotes*.

*Appendix B. presents the completed COREQ checklist*.

*Appendix C. presents an example of the initial coding tree generated from the data*.

## Data Availability

The datasets generated and analysed during the current study are not publicly available due to the risk of exposing the identity of participants, but it can be available from the corresponding author on reasonable request.

## References

[1] Cluley V , Ziemann A , Feeley C , Olander EK , Shamah S , Stavropoulou C . Mapping the role of patient and public involvement during the different stages of healthcare innovation: a scoping review. Health Expect. 2022;25(3):840‐855. 10.1111/hex.13437 35174585 PMC9122470

[2] Baines RL , Regan de Bere S . Optimizing patient and public involvement (PPI): identifying its “essential” and “desirable” principles using a systematic review and modified Delphi methodology. Health Expect. 2018;21(1):327‐335. 10.1111/hex.12618 28929554 PMC5750770

[3] Greenhalgh T , Hinton L , Finlay T , et al. Frameworks for supporting patient and public involvement in research: systematic review and co‐design pilot. Health Expect. 2019;22(24):785‐801. 10.1111/hex.12888 31012259 PMC6737756

[4] Corporate Consultation Secretariat, Health Policy and Communications Branch . Health Canada Policy Toolkit for Public Involvement in Decision Making. Health Canada; 2000.

[5] Miah J , Dawes P , Edwards S , Leroi I , Starling B , Parsons S . Patient and public involvement in dementia research in the European Union: a scoping review. BMC Geriatr. 2019;19(1):220. 10.1186/s12877-019-1217-9 31412788 PMC6694462

[6] Gove D , Diaz‐Ponce A , Georges J , et al. Alzheimer Europe's position on involving people with dementia in research through PPI (patient and public involvement). Aging Ment Health. 2018;22(6):723‐729. 10.1080/13607863.2017.1317334 28513210

[7] Bethell J , Pringle D , Chambers LW , et al. Patient and public involvement in identifying dementia research priorities. J Am Geriatr Soc. 2018;66(8):1608‐1612. 10.1111/jgs.15453 30084194

[8] Snowball E , Fernandez Loughlin R , Eagleson H , et al. Engagement of people with lived experience of dementia advisory group and cross‐cutting program: reflections on the first year. Res Involv Engagem. 2022;8(1):28. 10.1186/s40900-022-00359-5 35752841 PMC9233803

[9] Scottish Dementia Working Group Research Sub‐Group, UK . Core principles for involving people with dementia in research: innovative practice. Dementia. 2014;13(5):680‐685. 10.1177/1471301214533255 24858551

[10] Beresford‐Dent J , Sprange K , Mountain G , et al. Embedding patient and public involvement in dementia research: reflections from experiences during the ‘Journeying through Dementia’ randomised controlled trial. Dementia. 2022;21(6):1987‐2003. 10.1177/14713012221106816 35670381 PMC12064857

[11] Bavelaar L , McCann A , Cornally N , et al. Guidance for family about comfort care in dementia: a comparison of an educational booklet adopted in six jurisdictions over a 15 year timespan. BMC Palliat Care. 2022;21(1):76. 10.1186/s12904-022-00962-z 35578219 PMC9112535

[12] Bavelaar L , Nicula M , Morris S , et al. Developing country‐specific questions about end‐of‐life care for nursing home residents with advanced dementia using the nominal group technique with family caregivers. Patient Educ Couns. 2022;105(4):965‐973. 10.1016/j.pec.2021.07.031 34376304

[13] Harding AJE , Doherty J , Bavelaar L , et al. A family carer decision support intervention for people with advanced dementia residing in a nursing home: a study protocol for an international advance care planning intervention (mySupport study). BMC Geriatr. 2022;22(1):822. 36289458 10.1186/s12877-022-03533-2 PMC9607827

[14] Thorne SE . Interpretive Description: Qualitative Research for Applied Practice. 2nd ed. Routledge; 2016:96.

[15] Tong A , Sainsbury P , Craig J . Consolidated criteria for reporting qualitative research (COREQ): a 32‐item checklist for interviews and focus groups. Int J Qual Health Care. 2007;19(6):349‐357. 10.1093/intqhc/mzm042 17872937

[16] Braun V , Clarke V . Reflecting on reflexive thematic analysis. Qual Res Sport Exercise Health. 2019;11(4):589‐597. 10.1080/2159676X.2019.1628806

[17] Byrne D . A worked example of Braun and Clarke's approach to reflexive thematic analysis. Qual Quant. 2022;56(3):1391‐1412.

[18] Braun V , Clarke V . Successful Qualitative Research: A Practical Guide for Beginners. Sage; 2013.

[19] Dedoose . Dedoose Version 9.0.84, Web Application for Managing, Analyzing, and Presenting Qualitative and Mixed Method Research Data. Sociocultural Research Consultants LLC; 2021. www.dedoose.com

[20] van der Steen JT , Hertogh CMPM , de Graas T , Nakanishi M , Toscani F , Arcand M . Translation and cross‐cultural adaptation of a family booklet on comfort care in dementia: sensitive topics revised before implementation. J Med Ethics. 2013;39(2):104‐109. 10.1136/medethics-2012-100903 23144015

[21] Markle‐Reid M , Ganann R , Ploeg J , et al. Engagement of older adults with multimorbidity as patient research partners: lessons from a patient‐oriented research program. J Multimorb Comorb. 2021;11:263355652199950. 10.1177/2633556521999508 PMC7975523 33796472

[22] Ganann R , McAiney C , Johnson W . Engaging older adults as partners in transitional care research. Can Med Assoc J. 2018;190(suppl):S40‐S41. 10.1503/cmaj.180396 30404851 PMC6472453

[23] Capstick A , Dennison A , Oyebode J , et al. Drawn from life: cocreating narrative and graphic vignettes of lived experience with people affected by dementia. Health Expect. 2021;24(5):1890‐1900. 10.1111/hex.13332 34378295 PMC8483207

[24] Di Lorito C , Godfrey M , Dunlop M , et al. Adding to the knowledge on patient and public involvement: reflections from an experience of co‐research with carers of people with dementia. Health Expect. 2020;23(3):691‐706. 10.1111/hex.13049 32181553 PMC7321727

[25] Low LF , Purwaningrum F . Negative stereotypes, fear and social distance: a systematic review of depictions of dementia in popular culture in the context of stigma. BMC Geriatr. 2020;20(1):477. 10.1186/s12877-020-01754-x 33203379 PMC7670593

[26] Sublett JF , Bisconti TL . Benevolent ageism: the correlated of overaccommodation towards older adults. Innov Aging. 2019;3:S970‐S971.

[27] Dupuis SL , Gillies J , Carson J , et al. Moving beyond patient and client approaches: mobilizing ‘authentic partnerships’ in dementia care, support and services. Dementia. 2012;11(4):427‐452. 10.1177/1471301211421063

[28] Puts MTE , Sattar S , Ghodraty‐Jabloo V , et al. Patient engagement in research with older adults with cancer. J Geriatr Oncol. 2017;8(6):391‐396. 10.1016/j.jgo.2017.05.002 28599854

[29] Pitimson N . Teaching death to undergraduates: exploring the student experience of discussing emotive topics in the university classroom. Educ Rev. 2021;73(4):470‐486. 10.1080/00131911.2019.1646706

[30] Backhouse T , Kenkmann A , Lane K , Penhale B , Poland F , Killett A . Older care‐home residents as collaborators or advisors in research: a systematic review. Age Ageing. 2016;45(3):337‐345. 10.1093/ageing/afv201 26790454 PMC4846791

[31] Fortune D , McKeown J , Dupuis S , de Witt L . “It was like reading a detective novel”: using PAR to work together for culture change. J Aging Stud. 2015;34:38‐47. 10.1016/j.jaging.2015.04.002 26162724

[32] Glowinski B , Vellani S , Aboumrad M , et al. The Canadian long‐term care sector collapse from COVID‐19: innovations to support people in the workforce. Healthc Q. 2022;25:20‐26. 10.12927/hcq.2022.26982 36562580

[33] Dupuis S , McAiney CA , Fortune D , Ploeg J , Witt, L . Theoretical foundations guiding culture change: the work of the partnerships in Dementia Care Alliance. Dementia. 2014;15(1):85‐105. 10.1177/1471301213518935 24419355 PMC4674759

