# Supplementary material for: Patient and public involvement in international research: Perspectives of a team of researchers from six countries on collaborating with people with lived experiences of dementia and end‐of‐life
Source: Health Expect. 2023 Dec 24;27(1):e13942. doi: 10.1111/hex.13942 (PMC10748824; doi:10.1111/hex.13942)
Supplement: Supplementary file 1 — Appendix A. Additional illustrative quotes. [file HEX-27-e13942-s002.docx]

**Appendix A**. Additional Illustrative Quotes

| **Themes** | **Subthemes** | **Illustrative Quotes** |
| --- | --- | --- |
| **1- Groundwork to engage in research, people with lived experience in dementia and ^1^EOL care** | 1. Advocacy for engaging experts by experience | “I think that it is crucial that you have PPI, you have that involvement throughout the research, because researchers, we may be great at putting proposals together and developing everything, but we could be completely off of what is actually needed. And it’s the PPI involvement that brings it back to reality and brings us back into what is key, what is pivotal for them…they will keep our perspective correct and make sure that we are recognizing their needs and ensure that what we are providing is in the correct language, in the correct format ...” (Participant 2) |
|  | 1. Consideration for cultural diversities across countries | “The idea of a hierarchy between patients and healthcare providers and … the level of paternalism from healthcare providers is different in the different countries. But maybe it’s not. I’m not informed. I would say that in some countries people may be more willing to say what they think than in others. And then I also think…if it’s supposed to be uniform in all of these countries…. I suspect that what is comfortable for families in some places may be more comfortable in some places and less comfortable in other places. And then maybe having discussions among family members from those different countries will bring that to light. And maybe other people might have assumptions like what I’m making when there really aren’t differences or whatever.” (Participant 15)  “I think it can really help the research to be useful at the end. So, I like the idea very much. It's just that in our country it's not very usual to involve patients or families like that, so I look forward to this experience as part of this project. Like from the university experience which we have, we get a lot of feedback from patients and families that they are interested in what we are doing, that they are interested in what the results will change, and how can they be extra helpful. So, I think that if we offer the opportunity to be involved in the whole process, I can imagine that our people would be grateful for that chance, at least that's my patients.” (Participant 24) |
|  | 1. Partnership with organizations | “If it’s a patient advocacy organization, … and are more involved in policy and planning for dementia. I think having those organizations is really important. They can give that wider context of what is going on nationally or internationally. Maybe like funding streams, that might be important to consider. It adds credibility to the results if you can have the support of all these organizations and say that the [x organization] has been involved in this research from the start. They helped to direct the research, looked at the results and endorsed the results. I think that adds huge credibility.” (Participant 31) |
|  | 1. Carefully cultivated research team | “So you're not looking for someone here who has, you know, a higher degree, you know, research. I mean they might have that but they have to be someone who's a high degree of emotional intelligence, but also someone who's used to talking to people with dementia, someone who's used to talking to family carers, ideally someone who has a high degree of awareness of the sensitivities involved.” (Participant 32)  “I think training in communication skills is a big thing. It’s really important to be able to communicate sensitively and appropriately when you are dealing with individuals who have dementia and also when you are dealing with the family carers, because at the end of the day, the things you are going to be talking about are quite sensitive issues and it can be quite a distressing time. So I think that’s a key thing that I have previously found really beneficial.” (Participant 5) |
|  |  |  |
| **2- Planning for research activities is key** | 1. Thoughtful process of engaging people with lived experiences | “I think, it will be difficult because if these patients really have advanced dementia, then it's ... I don't think they are really capable of giving advice anymore. Or you have to time the meetings in such a way that people are clear at that moment which I think is a bit difficult to do. And, at the same time, if you have people with early stage dementia, I wonder how representative they really are of the target population. So I think advanced dementia involvement in this maybe not possible but people with early stage dementia maybe.” (Participant 22)  “I think it's really important to learn from family carers who've actually gone through the entire process from diagnosis through to advanced dementia through to end-of-life care ... They will have learnt from the whole trajectory, so not just the care planning but also when care planning went wrong, when there were crises, when there were inappropriate hospital admissions, when there was inappropriate prescribing around final weeks or days of life. So, you know, miscommunication between the health care team or the social care team or the nursing home. So I think, if possible, one wouldn't just have family carers of people with dementia who are in nursing homes, but I think one would have family carers of people with dementia who'd passed away.” (Participant 32) |
|  | 1. Consideration for accessibility and accommodation | “So instead of just saying, for example, we are going to have a teleconference call and here’s the number and here is where you login and talk to you on Tuesday, really needing to think through is the person with dementia comfortable talking with a computer and speaking on the telephone?... what kind of support might be needed and embedded in that activity that perhaps we might not think of if our day-to-day routine involves teleconference calls.” (Participant 18)  “I think sometimes when we're talking about things like mortality rates and, scores of different instruments that predict death, you know, I think you have to remember when you have patients and family members or caregivers at that table that those words have a different impact or could be potentially triggering, potentially upsetting. So I think that's one thing, particularly with end of life and, you know, dying and death and cut-off points and predictive things, where there's patients or carers or the general public the team needs to be really cognizant of” (Participant 13).  “I think you have to do it slowly and I think it's going to take a bit more time. I think everything ... you know you have to avoid research jargon, you have to let people be briefed beforehand. I think that ... and then at the meetings, the meetings have to be set up in a certain way so that people get a chance to speak and to contribute because otherwise the more confident people, for instance, people involved in advocacy organizations, will have a lot more to say. So I think it's, it's teasing out those things and giving it time really.” (Participant 32) |
|  | 1. Remunerate for time, knowledge, and expertise | “There is really powerful research that suggests that it’s not so much the money or the actual incentive. It may be for some people. But it mostly acts as a kind of symbol of reciprocity. So it’s a way of saying I’m not just using you up. I’m interested in a reciprocal arrangement so I’m acknowledging your time with this token. And I’m telling you why you are important and how you might contribute and why I value your contributions. So I think together that creates a kind of reciprocity.” (Participant 10) |
|  |  |  |
| **3- Focus on meaningful engagement** | 1. Involve with purpose and avoid tokenism | “I have a little bit of skepticism about that … to go to a board meeting where everybody else doesn’t have a mental illness, has money, is dressed in a certain way, knows how board meetings work, knows what Roberts Rules are or this kind of stuff. If you don’t adapt so that people can be meaningfully involved, then they are just there but they are not really having a say. And I’ve been on the board of my local Alzheimer’s Society and there are representatives on the board who are families who have had someone with dementia. And I’m not sure that they actually go out of their way to see what their view is on something or to orient them to how these meetings work or maybe adapt the meetings to be more friendly towards a family member who maybe doesn’t come from the business world and knows how board meetings work. So I’ve seen places where they are there in name, but not really…” (Participant 15)  “…some researchers, they may never have had any contact with someone with a health condition or a more vulnerable person. I think it's a really different way of working with PPI anyway and it's very much about seeing people as partners and I think a lot of people tend to think it's just more about consultation and just that you would do an interview. But it's different, they're a partner and they have an equal voice and an equal say.” (Participant 11). |
|  | 1. Garnering trust in the research relationship | “I think having people with dementia and family caregivers is really important because it actually highlights to people that their opinions and their experiences are actually valuable. That we are not doing kind of a snatch and grab, running in and grabbing their information and running off with it. And that they have no contribution to make. I think it’s really important to convey the message that family caregivers, people with dementia have a lot to contribute to knowledge as well, beyond just being passive participants. I think it’s really important to make sure their voice is being heard.” (Participant 14) |
|  | 1. Strive to create guidance for future research engagement | “My concern is how do we make sure that they are engaged like other regular academic co-applicants and do not feel marginalized in the process? Then, then the question is in terms of assembling sort of PPI representation from an advisory panel perspective, do we do that at the country level? You know does each country have its own PPI advisory committee? And how big or small should they be? Who should be sitting on those types of advisory panels? And then do we need to have an international/transnational advisory panel as well? Do we have the budget resources to sort of maintain or realize our aspirations for real PPI engagement? How do we use them? do we have the resources to use them, to maximize their use? So I'm hoping it'll come out of the exercise which we're doing right now is that we'll get some guidance in terms of what that structure and process may look like.” (Participant 1) |
|  |  |  |
| **4- Having foresight for practical issues shaping engagement of people with lived experiences** | 1. Awareness of vested interests of diverse people | “People engage in this for different reasons. They have different motivations and different talents, and it might be important to do some preliminary assessment or screening to figure out what a person's motivations are and what they're likely to lend the project” (Participant 26) |
|  | 1. Watch out for emotional needs and vulnerabilities of partners | “There were emotional issues, because I asked family carers to remember the last period of their relative’s life. And so it was emotionally challenging for them to remember. So the interview had to be stopped several times to give them the time to acquire again a balance of their feelings.” (Participant 29) |
|  | 1. Mitigate challenges of retention | “I think the difference between caregivers of people with dementia and other caregivers would be that this is talking about end of life care. So I guess it depends where the person with dementia is on the trajectory of the dementia journey and where the caregivers are on the trajectory of that dementia journey, which they may all be in a different place. So I think that may be a challenge as to whether or not carers want to think that far ahead. And whether or not you might be looking at carers who have been through end of life care and who at this point, might be prepared to be involved in the study. But then the problem with that would be that a lot of caregivers once their person with dementia dies tends to try to get on with their lives and then move away from this. So I think there are sort of challenges.” (Participant 3) |
