# Supplementary material for: Patient and public involvement in international research: Perspectives of a team of researchers from six countries on collaborating with people with lived experiences of dementia and end‐of‐life
Source: Health Expect. 2023 Dec 24;27(1):e13942. doi: 10.1111/hex.13942 (PMC10748824; doi:10.1111/hex.13942)
Supplement: Supplementary file 3 — Appendix C. presents an example of the initial coding tree generated from the data. [file HEX-27-e13942-s003.docx]

**Additional file 2. Initial coding tree**

|  |
| --- |
| Barriers to patient and public involvement in research |
| Caregivers' belief that residents cant participate/caregivers overprotective |
| Cognitive decline as a barrier |
| Concerns with integrity of research |
| Difficulties with recruitment versus downside of engagement |
| Maintaining interests and partnership |
| Motivation facilitates participation |
| Diversity of participants can be a barrier/mixing scholars and people with lived experience  Developing a research team |
| Dynamics of participants in PPI group |
| Emotional distress/response (sensitivity) to topics |
| lack of awareness is a barriers |
| Lack of knowledge as a barrier/public requires training |
| Language as barrier |
| Older adults dont believe in their capacity to participate |
| Policy makers and executives as research/citizen participants |
| PPI can be time consuming for the researcher |
| Staff s believe that residents cant participate |
| Staff protective of residents/family (deter them from participating) |
| Structural barriers to participation |
| Technology |
| Time constraints serving as a barrier |
| Differences in motivation to participate between researchers and citizen/PLED |
| Facilitators to patient and public involvement in research |
| Facilitating engagement |
| Feeling valued/of value |
| Lip service versus participation for making a difference |
| Motives for participation |
| Demand from the funding bodies |
| Upliftment of care quality |
| Patients involvement in research encourages benefits in health care |
| Patients making a difference |
| Organizational culture |
| Outlined objectives for patient and public involvement |
| Trust between carers and staff |
| Organizations as partners |
| Participants with prior research experience |
| Patient replacements/advisors/representatives in research |
| Challenge in finding those with lived experiences |
| Openness to share personal views/willingness to share |
| Reimbursements and remunerations |
| Ethical questions |
| Goals of engagement/PPI in research activities |
| Create guidelines on PPI for countries it is not prevalant |
| How tos of engagement/faces of meaningful participation |
| Consider accessibility when planning PPI in research |
| Create space to listen to them |
| Ease down jargons |
| Orientation/setting expectation at the outset |
| planning a uniform approach |
| Setting expectations at the outset |
| Training citizens on how to engage in research or advisory activities |
| What is the role of PLE |
| Researchers' attributes |
| Awareness of/cognizant of their emotional needs |
| Encouragement of patient and public involvment in research |
| Garnering trust in the research relationship |
| Participants versus partner |
| Past experience with research/roles played by PPI/roles allocated to participants by researchers |
| not experienced with research/PPI |
| Past experience of interviewee |
| Past experience with PPI  Prerequisites for PPI |
| Researcher with lived experience |
| Researchers' learning edges |
| Support provided by researcher |
| Training for role in study with PPI |
| Understanding of palliative care/EOL (background knowledge) |
| Vested interests and individual agendas |
| Value in patient/care partner engagement |
| Engagement of people with lived experience make research relevant |
| Engagement of policy maker makes research practical |
| Hopes from participants with lived experiences (patients/care partners) |
| Being able to describe from own lived experience |
| Considerations for cultural diversities |
| Objectiveness in assessing effectiveness of PPI |
| Protocol versus personal experience |
